# Antibiotic use and resistance: a cross-sectional study exploring knowledge and attitudes among school and institution personnel in Tbilisi, Republic of Georgia

**DOI:** 10.1186/s13104-015-1477-1

**Published:** 2015-09-29

**Authors:** Ketevan Kandelaki, Cecilia Stålsby Lundborg, Gaetano Marrone

**Affiliations:** AIETI Medical School, Chonkadze St. N24, 0114 Tbilisi, Georgia; Global Health (IHCAR)/Department of Public Health Sciences, Karolinska Institutet, Tomtebodavägen 18A, 17177 Stockholm, Sweden; Chonkadze St. N24, 0114 Tbilisi, Georgia

**Keywords:** Antibiotics, Antibiotic resistance, Prescription, Public health, Policy, Georgia

## Abstract

**Background:**

The Republic of Georgia lacks regulations regarding drug prescriptions. In pharmacies, all drugs except psychotropic medication are sold legally without prescription anti-, including anti-tuberculosis agents. Due to the lack of adequate policies and regulations, the big share of responsibility regarding antibiotic education lies with the general public. This study examines public knowledge and attitudes toward antibiotic use and resistance in the Republic of Georgia among personnel from government schools and other public institutions.

**Methods:**

This cross-sectional study was conducted in June 2011 using a quantitative questionnaire. Convenience sampling method was used. Participants included 250 individuals aged 21–80 years, from government schools and public institutions. Participants were from Tbilisi as well as the surrounding rural and urban areas. Respondents provided demographic data along with statements on knowledge and attitudes towards antibiotic use and resistance. Poisson and logistic regression models were used to study the relationship between knowledge, attitude outcomes and socio-demographic characteristics.

**Results:**

The overall response rate was 75 % (n = 187), of which 80 % were female. Approximately 91 % of respondents had used antibiotics at least once and 55 % agreed that antibiotics speed up recovery from common colds. A number of respondents (55 %) reported having received antibiotics without previously consulting a doctor and 62 % reported having purchased antibiotics without a prescription. Respondents demonstrated some misunderstanding around the terms ‘bacteria’ and ‘virus.’ About 52 % of participants agreed that antibiotics are effective against bacteria; however, 55 % also agreed that antibiotics are effective against viruses. Trust in doctors was high at 80 %. More knowledge was associated with a lower probability of having purchased antibiotics without medical consultation.

**Conclusions:**

The study findings demonstrate that respondents have several misconceptions and lack knowledge on proper antibiotic use and resistance. High proportion of people use antibiotics without a medical prescription or consultation, while having high trust in the medical personnel. We believe that the high level of trust in doctors shown by our respondents should be acknowledged by the Georgian government, health care providers and public health policy professionals. Furthermore, the information should be utilized in future educational and antibiotic resistance awareness raising campaigns.

**Electronic supplementary material:**

The online version of this article (doi:10.1186/s13104-015-1477-1) contains supplementary material, which is available to authorized users.

## Background

Antibiotic resistance can be defined as the ability of a microorganism to survive and resist exposure to antimicrobial drugs, threatening the effectiveness of successful treatment of infection. Resistance can be transferred genetically from one microorganism to another. Antibiotic resistance is a recognized public health issue at the local, national and global levels. Currently antibiotic resistance is a concern as it is no longer a predictor of maintaining health within populations but an increasing threat to future of health as antibiotics are being misused [1–5].

Prevalence of antibiotic resistance varies among countries but in general it is positively correlated with prescribed outpatient antibiotic use at the national level [6, 7]. However, Antibiotic consumption can also include self-medication, (i.e. antibiotic use without prescription) [8]. This occurs through the use of leftover antibiotics from previously prescribed courses, antibiotics obtained from relatives or friends or bought without prescription, and antibiotics obtained both legally and illegally.

Resistance is a severe problem in the treatment of tuberculosis (TB). In 2010, multi-drug-resistant TB (MDR-TB) was estimated at 3.4 % of all new TB cases worldwide. Georgia is one of the 18 high-priority countries with regards to TB with a 2008 prevalence rate of 135.5 cases/100,000 people, of which 6.4 % were TB cases in children under 15 years of age [9]. While in the same year, among European Union (EU) and European Economic Area (EEA) counties, the lowest TB prevalence rate was seen in Iceland at 1.9/100,000, and among non- EU/EEA countries, Andorra’s prevalence rate was estimated at 4.7/100,000 [9]. In 2008, the drug resistance surveillance showed 20 % multi-drug resistant TB cases of which 4 % were extensively drug resistant (EDR) [9]. Misuse and overuse of anti-TB drugs, inappropriate treatment regimens, adherence failure and interrupted drug supplies are risk factors for further emergence of MDR-TB in Georgia [10].

Georgia is a former Soviet Union Republic, which gained its independence on April 9th, 1991 [11]. In 2011, Georgia had an estimated population of approximately 4.4 million people [12]. Tbilisi, the capital, is the largest urban area with a population of about 1.5 million. Since gaining its independence, the nation has suffered intense civil conflicts. As a result, a large influx of internally-displaced people (IDP) (approximately 300,000) has migrated to major cities in Georgia (including up to 22,000 people displaced in August 2008 after the Russian–Georgian war [13]). The events of the last two decades have worsened the country’s economic situation. While Georgia’s Human Development Index (HDI) ranked 75 in 2011 (out of 187 countries in total), the socioeconomic situation in the country is difficult, as more than 16 % of Georgia’s population is unemployed and 10 % fall below the national poverty line [14, 15]. After gaining independence, its healthcare system and financing moved towards program-based financing [11]: the government pays only 20–30 % of Georgia’s total health expenditure, while the rest is covered by the population through out-of-pocket payment (OOPP) [11, 16, 17].

In Georgia, all drugs, except for psychotropic medication, are sold legally without prescription. Studies showed that even anti-TB drugs, including *isoniazid* and *rifampin* were widely available without prescription at pharmacies in Tbilisi [10].

Due to the above-described situation, approximately 95 % of the Georgian population tends to avoid visiting a physician, which has resulted in the habit of self-treatment [11]. Since most people who engage in self-treatment have little or no medical knowledge. Those individuals often experience medical side-effects and threaten their own health when using self-prescribed medication, including antibiotics and other drugs [11].

To date, there is no study documenting knowledge and attitudes towards antibiotic use in Georgia. Thus, the aim of this study has been to examine the knowledge and attitudes toward antibiotic use and resistance among school and public institution personnel.

## Methods

### Participants and sampling

In Georgia, people are often afraid to participate in research studies due to fear of exposure and confidentiality concerns. In order to have elicited participation in this study documenting knowledge and attitudes towards antibiotic use, considering the difficulty in accessing the public, we applied a convenience sampling method. We identified networks and contacts mostly in federally funded government institutions including schools, ministries and public service sectors in Tbilisi and the surrounding rural and urban areas. In 2011 eight schools and public institutions were approached. All individuals working at each school or institution, regardless of the type of their contract of employment (part-time, full-time etc.) where invited to participate in this study. Participants were eligible if they were above 21 years of age. Medical personnel (nurses, doctors) were intentionally excluded due to possible response bias and more knowledge on the topic.

### Data collection instrument and data collection

The questionnaire (Additional file 1: Appendix 1) was constructed based on similar research conducted in Sweden [18] but modified to suit the Georgian context. It was tested for face and content validity with persons not participating in the main study. The study respondents where verbally informed about the research by administrative unit representatives of each institution. The questionnaire was distributed to the participants together with an introductory letter and collected after 2 weeks from the especially secure post boxes at administrative units. The questionnaire contained five sections, covering questions about (1) socio-demographics and information about previous antibiotic use, (2) access to antibiotics (3) knowledge on areas of antibiotic use and effectiveness (4) knowledge on side-effects of antibiotic treatment and antibiotic resistance, and (5) expectations from doctors, doctors’ habits and the doctor-patient relationships. Sections (2)–(5) consisted of several statements to which the respondent could answer ‘yes’, ‘no’ or ‘I don’t know’. The socio-demographic characteristics collected included: gender (male/female), age (<30/30–50/>50), number of children in the household (0/1/2/3/>3, which later during the data analysis was dichotomized as none/one or more children), area of residence (Old Tbilisi/Vake-Saburtalo/Didube-Chugureti/Gldani-Nadzaladevi/Isani-Samgori), educational level (elementary/upper secondary/university-higher education), and type of education (health-related/not health-related).

### Data management and statistical analyses

Questionnaire data were entered in Excel by the first author and transferred to STATA 12 (Stata Corp. College Station, TX, USA) for analysis.

During data analysis*, awareness, resistance and general* scores were created to assess knowledge. The awareness score was calculated as a sum of the correct answers to the questions under sections (2) (three questions on access to antibiotics) and (3) (14 questions on knowledge about antibiotic use and its effects). Resistance scores summed up the correct answers for the questions from section (4) (15 questions on side effects and resistance), whilst the general score represented the sum of correct answers on the questions in all of the above mentioned sections. The Cronbach’s alpha has been calculated for each score.

Descriptive statistics were calculated using median and standard deviation for numerical variables, and frequencies and percentages for categorical variables. T tests, χ^2^ tests and correlation coefficients were used for bivariate analyses of the relationship between independent variables and the outcomes of interest.

For count outcomes such as, *awareness,resistance, and general* scores, stepwise Poisson regression models were used to study the relationship between scores and socio-demographic characteristics. Stepwise logistic regression models were performed to study the association between ‘having purchased Antibiotics without prescription’ and ‘having received Antibiotics without prior medical consultation’ (both binary variables), *versus* demographic characteristics (age, gender, health related training, children in household and living area) and knowledge scores (*awareness,resistance, and general* scores). For all the regression models, variables with a *p* ≤ 0.25 in the bivariate analyses were considered in the stepwise models (to be more conservative in the selection of variables), and *p* < 0.05 were considered significant in the final models.

### Ethical considerations

The Heads of the public institutions where the questionnaires were distributed were contacted prior to the study and were informed about the topic of the study. They gave their consent for their personnel to participate. Subsequently, potential participants were approached and given information about the study, and that results would only be presented at an aggregate level. Those who agreed gave their oral/written consent before completing the questionnaire. In Georgia, these kinds of studies do not require formal ethical permission.

## Results

### Description of the study participants

Two hundred and fifty participants were included in this study of which 187 completed the survey. The participants’ background characteristics are summarized in Table 1. Nearly half of the respondents belonged to the 30–50 age group. In terms of education, 92 % had a higher education degree.

**Table 1:** Background characteristics of the 187 respondents (personnel of government schools and institution, in Tbilisi and surrounding rural and urban areas) who responded to the questionnaire and knowledge scores

Characteristic	N (%)
Sex	
Female	150 (80)
Male	37 (20)
Age	
<30	39 (21)
30–50	91 (49)
>50	57 (30)
Education	
Elementary school	3 (2)
Upper secondary education	12 (6)
University/higher education	172 (92)
Number of children (3- to 6-year-old) in the household	
None	137 (73)
One	27 (14)
Two	16 (9)
Three or more	7 (4)
Living area	
Old Tbilisi	48 (26)
Vake Saburtalo	54 (29)
Didube Chugureti	19 (10)
Gldani Nadzaladevi	54 (29)
Isani Samgori	12 (6)
Have ever used antibiotics	
Yes	171 (91)
No	13 (7)
Don’t know	3 (2)
Resistance score (median, IQR)	6 (4–7)
Awareness score (median, IQR)	8 (5–10)
General score (median, IQR)	14 (9–17)

Table 2 summarizes responses of those that agreed with the selected statements regarding access to antibiotics, antibiotic use and its effect, antibiotic resistance and the doctor-patient relationship. More than half (55 %) of respondents have received antibiotics without previous medical consultation and 62 % have purchased antibiotics without prescription. About 55 % believed that antibiotics speed up recovery from common colds. Information dissemination and trust in medical personnel were evaluated using four questions on the topic: *doctor*–*patient relationship*. The majority of respondents (54 %) agreed with the statement that ‘pharmacy staff often tell you how antibiotics should be used’. Most reported trusting doctors’ decisions both for prescribing (76 %) and for not prescribing antibiotics (80 %).

**Table 2:** Percentage of respondents who agreed with the selected statements

Topic	Correctness of the statement (if applicable)	Number (%) agreeing with the statement
Access to antibiotics		
Leftover antibiotics are good to keep at home in case of future need	Incorrect	91 (47)
Have received antibiotics without consultation with doctor		103 (55)
Have purchased antibiotics without prescription		115 (62)
Areas of antibiotic use and effectiveness		
Antibiotics speed up recovery from a cold	Incorrect	103 (55)
Antibiotics are effective against bacteria	Correct	97 (52)
Antibiotics are effective against viruses	Incorrect	102 (55)
Inflammation of the ear in a 3- to 6-year-old child almost always needs to be treated with antibiotics	Incorrect	54 (29)
Side-effect of antibiotic treatment and antibiotic resistance		
If you feel better after half the treatment with antibiotics you can stop taking them	Incorrect	40 (21)
Humans can become resistant to antibiotics	Incorrect	116 (62)
Bacteria can become resistant to antibiotics	Correct	102 (55)
Doctor–patient relationship		
Doctors often take time to inform the patient during the consultation how antibiotics should be used		114 (62)
Pharmacy staff often tell you how antibiotics should be used		100 (54)
I trust the doctor’s decision when s/he prescribes antibiotics		141 (76)
I trust the doctor’s decision if s/he decides not to prescribe antibiotics		148 (80)

### Regression analyses

Knowledge scores were quite low. The median resistance score, based on 17 questions, equaled six, and median awareness score, based on 15 questions, did not exceed eight. According to the Cronbach’s alpha analyses the resistance, awareness and general scores have an acceptable level of reliability (Table 3). Multiple Poisson regression analyses (Table 4) showed that the expected resistance score value is expected to be higher for the 30–50 and >50 age groups compared to the <30 age group. Significantly higher values of awareness and general scores were also seen for the 30–50 age group as compared to the <30 age group. Male respondents tended to have significantly lower resistance, awareness and general scores in comparison to female respondents. Awareness and general score counts were lower among respondents without any health-related training as compared to those who received some type of health-related training. Respondents with one or more children in the household have an expected higher general score compared to individuals without children. The expected number of the correct answers on the general score was lower among respondents living in the areas of Didube-Chugureti and Gldani-Nadzaladevi as compared to those living in the Old Tbilisi district.

**Table 3:** Reliability statistics, Cronbach’s alpha calculate for knowledge scores

Cronbach’s alpha	
Resistance score	0.69
Awareness score	0.69
General score	0.79

**Table 4:** Multiple Poisson regression: factors associated with resistance, awareness and general score

Independent variable	Category	Dependent variable		
		Resistance score N = 185	Awareness score N = 187	General score N = 185
		Coef. (95 % CI)	Coef. (95 % CI)	Coef. (95 % CI)
Age group	<30	Ref.	Ref.	Ref.
	30–50	0.19 (0.02; 0.36)*	0.14 (0.00; 0.28)*	0.18 (0.07; 0.29)***
	>50	0.21 (0.03; 0.39)*	0.05 (−0.11; 0.20)	0.10 (−0.02; 0.22)
Gender	Female	Ref.	Ref.	Ref.
	Male	−0.25 (−0.41; −0.08)**	−0.20 (−0.34; −0.05)**	−0.22 (−0.33; −0.10)***
Health-related training	Yes	Ref.	Ref.	Ref.
	No		−0.17 (−0.30; −0.04)**	−0.14 (−0.24; −0.05)****
Children in household	None	Ref.	Ref.	Ref.
	One or more			0.08 (−0.01; 0.17)
Area of residence	Old Tbilisi	Ref.	Ref.	Ref.
	Vake Saburtalo			−0.06 (−0.17; 0.05)
	Didube Chugureti			−0.21 (−0.36; −0.06)**
	Gldani Nadzaladevi			−0.15 (−0.26; −0.04)**
	Isani Samgori			−0.08 (−0.26; 0.11)

* p < 0.05, ** p < 0.01, *** p < 0.001

**** The parameters of the Poisson regression models can be interpreted as the difference in the logs of expected counts for a one unit increase in the predictor variable, given that the other predictor variables in the model are held constant. A positive coefficient indicates an increase in the expected count and a negative coefficient indicated a decrease

Logistic regression analyses showed that a higher awareness score is associated with lower odds of having purchased antibiotics without a medical consultation (p = 0.007) (Table 5). Age is positively correlated with odds of receiving antibiotics without consulting a doctor. The resistance score positively correlated with the odds to have purchased antibiotics without prescription.

**Table 5:** Logistic regression: factors associated with having received/purchased antibiotics without medical consultation or prescription

Independent variable	Category	Dependent variable	
		Received antibiotics without medical consultation	Purchased antibiotics without medical prescription
		OR (95 % CI)	OR (95 % CI)
Age group	<30	Ref.	
	30–50	4.32 (1.88; 9.90)**	
	>50	2.85 (1.20; 6.79)*	
Awareness score		0.87 (0.78; 0.96)**	0.90 (0.80; 1.01)
Resistance score			1.17 (1.01; 1.34)*

* p < 0.05, ** p < 0.01

## Discussion

This is the first study documenting knowledge and attitudes towards antibiotic use in Georgia. The topic has, however, been researched in similar studies in other countries such as Sweden, South Korea, Malaysia, the Denver metropolitan area, New Zealand, Netherlands and Tanzania [18–24]. Poor awareness and education regarding antibiotic use and resistance worldwide are some of the main challenges that need to be addressed at both the global and national levels in order to ensure and improve proper antibiotic use [2].

The participants in our study were not well-informed about antibiotics and their use, and the importance of antibiotic resistance and the spread of antibiotic-resistant bacteria. Our results show that more than half (55 %) of the respondents agreed that common colds are cured more quickly with antibiotics, which is more than double that reported in a similar Swedish study (19.1 %) [18]; in this study 55 % believed that antibiotics are beneficial against viruses compared to 21 % seen in Swedish study [18]. Misinterpretation of an individual’s own health status was also common in the general public where many people believed that most infections, regardless of etiology, respond to antimicrobials, and thus they use antibiotics for any perceived infection [25]. In primary care, respiratory tract infections account for one half to two-thirds of prescriptions [6, 26, 27]. Upper respiratory tract infections (URTI), commonly caused by viruses, are frequent reasons for medical consultation. Although URTI do not require antibiotics, they account for big portion of all antibiotic use in general practice [2, 28]. Recent guidelines suggest that in otherwise healthy individuals sometimes a ‘wait-and-see’ policy without immediate use of antibiotics is an option for certain URTI. Indeed delayed prescriptions may ease patient fears while simultaneously reducing inappropriate antibiotic use [29].

One may argue that a possible reason for inadequate knowledge among this study’s participants could be due to the microbiological terms used (‘bacteria’ or ‘virus’). Respondents might lack sufficient knowledge to differentiate between the two groups of pathogens. It may perhaps be beneficial to explain the differences between these terms when prescribing or not prescribing antibiotics to patients.

Our study revealed overlap between the results: more than half of participants received antibiotics without medical consultation and over 60 % purchased antibiotics without prescription. This can be explained by the fact that the questionnaire did not provide a clear differentiation between how study participants understood and perceived the word ‘prescription’. More specifically, whether a regular paper received from a doctor, with the name of a drug on it is perceived as a prescription, or whether they solely refer to special forms as prescriptions (forms/notes provided by medical personnel). It should also be noted that the questionnaire did not provide the opportunity for study participants to identify the sources from which they received antibiotics without medical consultation; therefore respondents could have received antibiotics from friends, neighbors, etc.

Studies showed that broad-spectrum antibiotics use is common in Georgia, with co-amoxiclav accounting for 42.9 % of total antibiotic use in 2011 [30]. Relating this fact to our study findings, the knowledge on antibiotic use as well as the risk of antibiotic resistance development is poorly understood among our respondents, which further strengthens the need to enhance measures enforcing public health educational interventions targeting the general public. Public health educational interventions are considered to be one of the most successful strategies for antibiotic resistance control and prevention and are recommended in order to promote appropriate antibiotic use [31, 32].

Studies show that public knowledge of antibiotic treatment and antibiotic resistance awareness can influence patient demand for antibiotic prescriptions, as well as their behavior towards antibiotic use [33]. It should be acknowledged that prescription policies alone are not a universal solution for antibiotic resistance control and prevention. Studies suggest that medical prescriptions do not guarantee that a correct diagnosis has been made [24, 34, 35].

It is interesting to note that almost 80 % of participants reported trusting doctors’ decisions both for prescribing (76 %) and for not prescribing an antibiotic (80 %). Similar findings have been seen in a Swedish report, with relatively high trust seen towards doctors who do not prescribe antibiotics [18]. Although this study did not reveal a particular difference in trust when a doctor prescribed antibiotics or not, this high degree of trust, irrespective of the doctors’ decision, is important to note, since studies have shown that trust is not easily obtained through interventions. Trust in doctors is regarded as a fundamental component of the doctor-patient relationship and is related to patient satisfaction and adherence to treatment [36].

The majority of participants had correct knowledge of the importance of completing a full course of antibiotics even when symptoms improved. However, our questionnaire did not cover questions on whether individuals continue antibiotic treatment when they start feeling better. In order to avoid selection of resistant organisms, public health campaigns have been encouraging people to finish antibiotic treatment as prescribed, an approach which is somewhat questioned today. Although limited research has been done in this area, it has been seen that prolonged treatment might result in higher resistance rates, whilst shorter treatments could be more appropriate [37].

Notably, in government-funded institutions and public schools in Tbilisi, around 82 % of personnel are female [38], which explains why women made up 80 % of the respondents. Our results show that individuals with no health-related training, younger than 30-year-old, males, those without children in the household, and living in poorer parts of the city had limited knowledge on antibiotic use. These categories should be the main targets for future public health interventions.

Didube-Chugureti and Gldani-Nadzaladevi regions, districts with lower economic status, had lower expected counts of the *general score* compared to those living in the Old Tbilisi region, which is considered one of the more affluent areas. Further studies are required for better understanding of the geographical differences. We also found that people older than 30 years of age were less likely to declare having received antibiotics without medical consultation. In addition, study participants with more knowledge on antibiotic resistance are more prone to purchase antibiotics without prescription. Further research would be useful to establish a clear motive and to better understand what is meant and understood by ‘prescription’.

### Methodological considerations

In this study convenience sampling method was used. We therefore acknowledge the possibility of selection bias and that the results cannot be generalized to the whole Georgian population.

The lack of information on respondent’s income distribution limits our ability to determine whether socioeconomic status plays any role on behavior and attitudes towards antibiotic use. Respondents had the opportunity to take the questionnaires home. Consequently, they may have looked up the correct answers online or conferred with someone else before responding. If so, this may lead to some overestimation of knowledge and attitudes towards antibiotics compared to the general population. The consequence of this would be that the problems raised in this study may actually be more severe if the whole country is considered. Furthermore, the survey was conducted within school and governmental institutions, where almost all personnel had university-level education. In addition the study was based in the capital, Tbilisi, where the population’s socioeconomic status is relatively better in comparison to the rest of the country. We would like to note that while creating *awareness, resistance and general* scores, the scores were summed up without using any weighting, giving the same importance to each question.

Tbilisi also offers relatively better access to healthcare facilities and higher possibilities of affording healthcare services. Thus, access to antibiotics without prescriptions and without medical consultation is expected to be more widespread among the general public. The knowledge scores were created summing up the number of correct answers without weighting the answers according the estimated importance. It should be noted that the study was based on self-reported information and depends on the honesty and recall ability of the study’s participants, as well as their understanding of the questionnaire.

## Conclusions and recommendations

The findings demonstrate that respondents have several misconceptions and a lack of awareness on antibiotic use and resistance. Further qualitative and quantitative studies are needed to identify the determinants of attitudes, behavior, expectations and motivation that lead people to use and misuse antibiotics. Considering the complexity of infectious disease and antibiotic resistance management in the global healthcare arena, the Georgian government should commit to investing in public health education programmes for the public and healthcare professionals (including policymakers, doctors, nurses, pharmacists, and patients themselves). In addition, the government should require its healthcare system to develop proper regulations and prescription policies as well as controls for prescription drugs and empower the pharmacists’ role in raising awareness around the use of antibiotics and the growing antibiotic resistance within populations. Finally, public health strategies—including educational programmes—should be developed, targeting specific areas of misconception, misuse of antibiotics, and identification of at-risk populations in terms of improper antibiotic consumption.

## Additional file

10.1186/s13104-015-1477-1 Study questionnaire (in English and in Georgian); Appendix 1 includes the cross-sectional questionnaire used during the study.

## Abbreviations

EU: European Union
EEA: European Economic Area
HDI: Human Development Index
EDR: extensively drug resistant
MDR-TB: multi-drug resistant tuberculosis
TB: tuberculosis
URTI: upper respiratory tract infections
IDP: internally-displaced people
OOP: out-of-pocket payment
