# Supplementary material for: Antibiotic use and resistance: a cross-sectional study exploring knowledge and attitudes among school and institution personnel in Tbilisi, Republic of Georgia
Source: BMC Res Notes. 2015 Sep 29;8:495. doi: 10.1186/s13104-015-1477-1 (PMC4589112; doi:10.1186/s13104-015-1477-1)
Supplement: Supplementary file 1 — 10.1186/s13104-015-1477-1 Study questionnaire (in English and in Georgian); Appendix 1 includes the cross-sectional questionnaire used during the study. [file 13104_2015_1477_MOESM1_ESM.doc]

**Study Questionnaire (in English)**

**Serial number:...................**

**1. Introduction**

1.1 Gender

􀍙Female

􀍙Male

*Write down the first two antibiotic substances and the first two other substances*

1.2 Antibiotic substances: ______________________________________

1.3 Other substances: ______________________________________

1. 4 Have you ever heard of penicillin?

􀍙 Yes

􀍙 No

􀍙 Don.’t know

*Question 1.5!*

1.5 Have you ever used antibiotics?

􀍙 Yes

􀍙 No

􀍙 Don.’t know

*Jump to Question 1.8 if you have not used antibiotics, otherwise continue with Question 1.6*

1.6 How many times have you used antibiotics?

􀍙 Once

􀍙 Less than 10 times

􀍙 More than 10 times

1.7 When did you last use antibiotics?

􀍙 Within the last 12 months

􀍙 More than 12 months ago

􀍙 More than 10 years ago

1.8 How many children between 3 and 6 years old are there in the household?

􀍙 None

􀍙 One

􀍙 Two

􀍙 Three or more

*Jump to Question 1.12 if you don’t have any children between 3 and 6 years*

*old in the household.*

1.9 have any of your children been given antibiotics?

􀍙 Yes

􀍙 No

􀍙 Don.’t know

1.10 How many times has/have your child/ren been given antibiotics?

􀍙 Once

􀍙 Less than 10 times

􀍙 More than 10 times

1.11 When did your child/ren last receive antibiotics?

􀍙 Within the last months

􀍙 More than 12 months ago

1.12 What level of education have you achieved?

􀍙 Compulsory school

􀍙 Upper secondary education

􀍙 University-higher education

1.13 Living area/commune

􀍙 Old Tbilisi

􀍙 Vake Saburtalo

􀍙 Didube Chugureti

􀍙 Gldani Nadzaladevi

􀍙 Isani Samgori

1.14 Have you had any medical care education/training?

􀍙 Yes

􀍙 No

*Jump to Question 1.16 if the you have no medical care education or training*

1.15 What medical care education and training do you have?

_____________________________________________________________________

1.16 How old are you?

􀍙 <30

􀍙 30 - 50

􀍙 >50

1.17 Have you ever purchased antibiotics without prescription?

􀍙 yes

􀍙 no

1.18 Have you received antibiotics without consultation with doctor?

􀍙 yes

􀍙 no

**2. Access to antibiotics**

2.1 Left-over antibiotics are good to keep at home

in case they might be needed later on.

􀍙 Agree

􀍙 Don.’t agree

􀍙 Don.’t know

2.2 It.’s good to be able to get antibiotics from relatives

or friends without having to see a doctor.

􀍙 Agree

􀍙 Don.’t agree

􀍙 Don.’t know

2.3 It.’s good to be able to buy antibiotics

online, without having to see a doctor.

􀍙 Agree

􀍙 Don.’t agree

􀍙 Don.’t know

**3. Areas of use and effect**

3.1 Antibiotics are effective against bacteria.

􀍙 Agree

􀍙 Don.’t agree

􀍙 Don.’t know

3.2 Antibiotics are effective against viruses.

􀍙 Agree

􀍙 Don.’t agree

􀍙 Don.’t know

3.3 Colds are caused by bacteria.

􀍙 Agree

􀍙 Don.’t agree

􀍙 Don.’t know

3.4 Colds are caused by viruses.

􀍙 Agree

􀍙 Don.’t agree

􀍙 Don.’t know

3.5 Antibiotics speed up recovery from a cold.

􀍙 Agree

􀍙 Don.’t agree

􀍙 Don.’t know

3.6 If the nasal catarrh (snot) from a head cold is coloured, you

often need antibiotics to get rid of the cold.

􀍙 Agree

􀍙 Don.’t agree

􀍙 Don.’t know

3.7 If you have a cough for more than a week, you

often need antibiotics to get rid of the cough.

􀍙 Agree

􀍙 Don.’t agree

􀍙 Don.’t know

3.8 It.’s appropriate to use antibiotics when you have

a sore throat because otherwise you might catch something more serious.

􀍙 Agree

􀍙 Don.’t agree

􀍙 Don.’t know

3.9 It.’s appropriate to use antibiotics when you have tonsillitis

because otherwise you might catch something more serious.

􀍙 Agree

􀍙 Don.’t agree

􀍙 Don.’t know

3.10 Inflammation of the ear in a 3-6 year-old child

almost always needs to be treated with antibiotics.

􀍙 Agree

􀍙 Don.’t agree

􀍙 Don.’t know

3.11 Cystitis in a woman can also be cured

without antibiotics.

􀍙 Agree

􀍙 Don.’t agree

􀍙 Don.’t know

3.12 By using antibiotics you can avoid

having to be off-sick from work.

􀍙 Agree

􀍙 Don.’t agree

􀍙 Don.’t know

3.13 Different antibiotics are needed to

cure different diseases.

􀍙 Agree

􀍙 Don.’t agree

􀍙 Don.’t know

3.14 Antibiotics kill all the bacteria in the body.

􀍙 Agree

􀍙 Don.’t agree

􀍙 Don.’t know

**4. Side-effects and resistance**

4.1 If you get some kind of skin reaction when using an

antibiotic, you should not use the same antibiotic again.

􀍙 Agree

􀍙 Don.’t agree

􀍙 Don.’t know

4.2 Antibiotics can cause imbalance in the body.’s

own bacterial flora.

􀍙 Agree

􀍙 Don.’t agree

􀍙 Don.’t know

4.3 If you get side-effects during a course of antibiotics treatment

you should stop taking them as soon as possible.

􀍙 Agree

􀍙 Don.’t agree

􀍙 Don.’t know

4.4 If you feel better after half the treatment with antibiotics

you can stop taking them.

􀍙 Agree

􀍙 Don.’t agree

􀍙 Don.’t know

4.5 Use of antibiotics can reduce the bodies

own capacity to fight off infections.

􀍙 Agree

􀍙 Don.’t agree

􀍙 Don.’t know

4.6 Humans can be resistant to antibiotics.

􀍙 Agree

􀍙 Don.’t agree

􀍙 Don.’t know

4.7. The use of antibiotics can increase

the resistance of bacteria to them

􀍙 Agree

􀍙 Don.’t agree

􀍙 Don.’t know

4.8 Bacteria can be resistant to antibiotics.

􀍙 Agree

􀍙 Don.’t agree

􀍙 Don.’t know

4.9 The use of antibiotics can increase

the resistance of viruses to them.

􀍙 Agree

􀍙 Don.’t agree

􀍙 Don.’t know

4.10 Viruses can be resistant to antibiotics

􀍙 Agree

􀍙 Don.’t agree

􀍙 Don.’t know

4.11 The use of antibiotics among animals can

reduce the effect of antibiotics among humans.

􀍙 Agree

􀍙 Don.’t agree

􀍙 Don.’t know

4.12 Resistance can spread from animals to humans.

􀍙 Agree

􀍙 Don.’t agree

􀍙 Don.’t know

4.13 Resistance can spread from human to human.

􀍙 Agree

􀍙 Don.’t agree

􀍙 Don.’t know

4.14 Resistance is a problem in Georgia today.

􀍙 Agree

􀍙 Don.’t agree

􀍙 Don.’t know

4.15 Resistance is a problem in the rest of the world today.

􀍙 Agree

􀍙 Don.’t agree

􀍙 Don.’t know

**5. Doctors’ habits and the patient/doctor relationship**

5.1 Doctors often take time to consider

carefully whether antibiotics are needed or not.

􀍙 Agree

􀍙 Don.’t agree

􀍙 Don.’t know

5.2 Doctors often prescribe antibiotics because

the patient expects it.

􀍙 Agree

􀍙 Don.’t agree

􀍙 Don.’t know

5.3 I trust the doctor.’s decision

when s/he prescribes antibiotics.

􀍙 Agree

􀍙 Don.’t agree

􀍙 Don.’t know

5.4 Doctors often take time to inform

the patient during the consultation how antibiotics should be used.

􀍙 Agree

􀍙 Don.’t agree

􀍙 Don.’t know

5.5 Pharmacy staff often tell you how antibiotics

should be used..

􀍙 Agree

􀍙 Don.’t agree

􀍙 Don.’t know

5.6 I often know how to use antibiotics even

if I am told how to.

􀍙 Agree

􀍙 Don.’t agree

􀍙 Don.’t know

5.7 I often know myself if I need antibiotics

before I meet the doctor.

􀍙 Agree

􀍙 Don.’t agree

􀍙 Don.’t know

5.8 I trust the doctor.’s decision

if s/he decides not to prescribe antibiotics.

􀍙 Agree

􀍙 Don.’t agree

􀍙 Don.’t know

5.9 A doctor who doesn.’t prescribe antibiotics when

the patient thinks s/he should is a bad doctor.

􀍙 Agree

􀍙 Don.’t agree

􀍙 Don.’t know

კითხვარი:

1. ელექტრონული ფოსტა ______________________
2. სქესი________
3. განათლება, პროფესია__________
4. საცხოვრებელი უბანი________
5. დაასახელეთ ერთი ან ორი ანტიბიოტიკის სახელწოდება რომელიც გსმენიათ____________
6. დაასახელეთ ორი სხვა წამლის , პრეპარატის სახელწოდება__________
7. როდესმე თუ გსმენიათ პენიცილინის, ანტიბიოტიკის შესახებ?

􀍙 დიახ

􀍙 არა

􀍙 არ ვიცი

1. როდესმე თუ მიგიღიათ ანტიბიოტიკი?

􀍙 დიახ

􀍙 არა

􀍙 არ ვიცი

**იმ შემთხვევაში თუ არ გამოგიყენებიათ ანტიბიოტიკი გადადით პირდაპირ კითხვა #11-ზე. ხოლო თუ გამოგიყენებიათ ანტიბიოტიკი მაშინ ჩვეულებრივად გააგრძელეთ კითხვარის შევსება.**

1. რამდენჯერ მიგიღიათ ანტიბიოტიკი?

􀍙 ერთხელ

􀍙 10-ზე ნაკლებჯერ

􀍙 10-ზე მეტჯერ

1. ბოლოს როდის მიიღეთ ანტიბიოტიკი?

􀍙 ბოლო 12 თვის განმავლობაში

􀍙 ერთი წლის წინ

􀍙 10 წლის წინ

1. რამდენი ბავშვია თქვენს ოჯახში 3-6 წლის ასაკის?

􀍙 არცერთი

􀍙 ერთი

􀍙 ორი

􀍙 სამი და მეტი

**იმ შემთხვევაში თუ არ გყავთ 3-6 წლის ასაკის ბავში/ბავშვები მაშინ გადადით კითხვა #15-ზე . წინააღმდეგ შემთხვევაში ჩვეულებრივად განაგრძეთ კითხვარის შევსება.**

1. თქვენს ბავშვებს როდესმე თუ დასჭირებიათ და თუ როდესმე მიუღიათ ანტიბიოტიკი?

􀍙 დიახ

􀍙 არა

􀍙 არ ვიცი

1. რამდენჯერ მიუღიათ ანტიბიოტიკები თქვენს ბავშვს/ბავშვებს?

􀍙 ერთხელ

􀍙 10-ზე ნაკლებჯერ

􀍙 10-ზე მეტჯერ

1. ბოლოს როდის მიიღეს თქვენმა ბავშვებმა ანტიბიოტიკები?

􀍙 ბოლო 1 თვის განმავლობაში

􀍙 1 წლის წინ

1. შეგიძენიათ თუ არა ანიბიოტიკები რეცეპტის გარეშე?

􀍙 დიახ

􀍙 არა

1. მიგიღიათ თუ არა ანტიბიოტიკები ეტიმის კონსულტაციის არეშე?

􀍙 დიახ

􀍙 არა

1. გაქვთ თუ არა რაიმე სახის სამედიცინო ანათლება მიღებული, რაიმე სახის ტრენინგი ან კურსი გავლილი?

􀍙 დიახ

􀍙 არა

1. რა სახის სამედიცინო განათლება გაქვთ მიღებული?_____________________
2. რომელ ასაკობრივ ჯგუფს მიეკუთვნებით?

􀍙 <21

􀍙 21-30

􀍙 31-40

􀍙 41-50

􀍙 51-60

􀍙 61-70

􀍙 >70

1. მორჩენილი ანტიბიოტიკების შენახვა კარგია, იმ შემთხვევში თუ ვინიცობაა და ისევ დაგჭირდეთ ანტიბიოტიკების გამოყენება.

􀍙 ვეთანხმები

􀍙 არ ვეთანხმები

􀍙 არ ვიცი

1. კარგია ანტიბიოტიკების მიღება ახლობლებისგან, მეგობრებისგან, ექიმის ნახვის და კონსულტაციის გარეშე.

􀍙 ვეთანხმები

􀍙 არ ვეთანხმები

􀍙 არ ვიცი

1. კარგია ანტიბიოტიკების შესყიდვა/ გამოწერა ინტერნეტ ვებ გვერდების საშუალებით , ექიმის კონსულტაცის და ნახვის გარეშე.

􀍙 ვეთანხმები

􀍙 არ ვეთანხმები

􀍙 არ ვიცი

1. ანტიბიოტიკები ეფეკტურია ბაქტერიების წინააღმდეგ.

􀍙 ვეთანხმები

􀍙 არ ვეთანხმები

􀍙 არ ვიცი

1. ანტიბიოტიკები ეფეკტურია ვირუსების წინააღმდეგ.

􀍙 ვეთანხმები

􀍙 არ ვეთანხმები

􀍙 არ ვიცი

1. გრიპს, უბრალო გაციებას იწვევენ ბაქტერიები.

􀍙 ვეთანხმები

􀍙 არ ვეთანხმები

􀍙 არ ვიცი

1. გრიპს, უბრალო გაციებას იწვევენ ვირუსები.

􀍙 ვეთანხმები

􀍙 არ ვეთანხმები

􀍙 არ ვიცი

1. გრიპისა და გაციების დროს ანტიბიოტიკების მიღება აჩქარებს გამოჯანმრთელების პროცესს.

􀍙 ვეთანხმები

􀍙 არ ვეთანხმები

􀍙 არ ვიცი

1. ანტიბიოტიკების გამოყენება მიზანშეწონილია თუ გრიპის დროს თან დაერთო ცხვირიდან მოყბითალო შეფერილი გამონადენი.

􀍙 ვეთანხმები

􀍙 არ ვეთანხმები

􀍙 არ ვიცი

1. თუ კი ხველება გაგიგრძელდათ ერთ კვირაზე მეტ ხანს მაშინ ანიბიოტიკების მიღებაა საჭირო.

􀍙 ვეთანხმები

􀍙 არ ვეთანხმები

􀍙 არ ვიცი

1. ჯანმრთელობის მდგომარეობის გაუარესების თავიდან აცილების მიზნით, მიზანშეწონილია ანტიბიოტიკების მიღება ყელის ტკივილის დროს.

􀍙 ვეთანხმები

􀍙 არ ვეთანხმები

􀍙 არ ვიცი

1. ჯანმრთელობის მდგომარეობის გაუარესების თავიდან აცილების მიზნით, მიზანშეწონილია ანტიბიოტიკების მიღება ტონზილიტის/ გლანდების ანთების დროს.

􀍙 ვეთანხმები

􀍙 არ ვეთანხმები

􀍙 არ ვიცი

1. 3- 6 წლის ბავშვებში ყურის ანთების დროს თითქმის ყოველთვის არის აუცილებელი ანტიბიოტიკებით მკურნალობა.

􀍙 ვეთანხმები

􀍙 არ ვეთანხმები

􀍙 არ ვიცი

1. ცისტიტი - სარდის ბუშტის ანთება შეიძლება განიკურნოს ანტიბიოტიკების მიღების გარეშეც.

􀍙 ვეთანხმები

􀍙 არ ვეთანხმები

􀍙 არ ვიცი

1. ანტიბიოტიკების მირებიტ შესაძლებელია თავიდან ავიცილოთ ავადობის მიზეზით სამსახურის გაცდენა.

􀍙 ვეთანხმები

􀍙 არ ვეთანხმები

􀍙 არ ვიცი

1. სხავასხვა ანტიბიოტიკია საჭირო სხვადასხვა დაავადების სამკურნალოდ.

􀍙 ვეთანხმები

􀍙 არ ვეთანხმები

􀍙 არ ვიცი

1. ანტიბიოტიკები ანადგურებენ ყველა ბაქტერიას ორგანიზმში.

􀍙 ვეთანხმები

􀍙 არ ვეთანხმები

􀍙 არ ვიცი

1. თუ ანტიბიოტიკების მირების დროს გაგინვითარდათ კანის რაიმე სახის გაღიზიანება, მაშინ იგივე ანტიბიოტიკი აღარ უნდა მიიღოთ.

􀍙 ვეთანხმები

􀍙 არ ვეთანხმები

􀍙 არ ვიცი

1. ანტიბიოტიკების მირებამ შეიძლება ამოიწვიოს ორგანიზმის ბუნებრივი ფლორის დისბალანსი.

􀍙 ვეთანხმები

􀍙 არ ვეთანხმები

􀍙 არ ვიცი

1. თუ ანტიბიოტიკებით მკურნალობისას გაგინვითარდათ რაიმე სახის გვერდითი ეფექტი მაშინ უნდა შეწყვიტოთ ანტიბიოტიკების მიღება.

􀍙 ვეთანხმები

􀍙 არ ვეთანხმები

􀍙 არ ვიცი

1. თუ ანტიბიოტიკებით მკურნალობის არასრული კურსის/ ნახევარი კურსის მიღების შემდეგ უკეთ იგრძენთ თავი , შეგიძლიათ შეწყვიტოთ მკურნალობა.

􀍙 ვეთანხმები

􀍙 არ ვეთანხმები

􀍙 არ ვიცი

1. ანტიბიოტიკების მიღებან შეიძლება შეამციროს ორგანიზმის უნარი თავად ებრძოლოს ინფექციებს.

􀍙 ვეთანხმები

􀍙 არ ვეთანხმები

􀍙 არ ვიცი

1. ადამიანი შეიძლება რეზისტენტული იყოს ანტიბიოტიკების მიმართ.

􀍙 ვეთანხმები

􀍙 არ ვეთანხმები

􀍙 არ ვიცი

1. ანტიბიოტიკების მიღებამ შეიძლება გაზარდოს ანტიბიოტიკებისადმი ბაქტერიების რეზისტენტობა.

􀍙 ვეთანხმები

􀍙 არ ვეთანხმები

􀍙 არ ვიცი

1. ბაქტერია შეიძლება რეზისტენტული იყოს ანტიბიოტიკების მიმართ.

􀍙 ვეთანხმები

􀍙 არ ვეთანხმები

􀍙 არ ვიცი

1. ანტიბიოტიკების მიღებამ შეიძლება გაზარდოს ანტიბიოტიკებისადმი ვირუსების რეზისტენტობა.

􀍙 ვეთანხმები

􀍙 არ ვეთანხმები

􀍙 არ ვიცი

1. ვირუსი შეიძლება რეზისტენტული იყოს ანტიბიოტიკების მიმართ.

􀍙 ვეთანხმები

􀍙 არ ვეთანხმები

􀍙 არ ვიცი

1. ცხოველებში ანტიბიოტიკების გამოყენებამ შეიძლება შეამციროს ანტიბიოტიკების ეფექტურობა ადამიანებში.

􀍙 ვეთანხმები

􀍙 არ ვეთანხმები

􀍙 არ ვიცი

1. რეზისტენტობა შეიძლება გავრცელდეს ცხოველიდან ადამიანზე.

􀍙 ვეთანხმები

􀍙 არ ვეთანხმები

􀍙 არ ვიცი

1. რეზისტენტობა შეიძლება გავრცელდეს ადამიანიდან ადამიანზე.

􀍙 ვეთანხმები

􀍙 არ ვეთანხმები

􀍙 არ ვიცი

1. რეზისტენტობა დღეს საქართველოს პრობლემაა.

􀍙 ვეთანხმები

􀍙 არ ვეთანხმები

􀍙 არ ვიცი

1. რეზისტენტობა დღეს მსოფლიოს პრობლემაა..

􀍙 ვეთანხმები

􀍙 არ ვეთანხმები

􀍙 არ ვიცი

1. ანტიბიოტიკების დანიშვნისას ექიმი საკმაო დროს უთმობს გადაწყვეტილების მიღებას.

􀍙 ვეთანხმები

􀍙 არ ვეთანხმები

􀍙 არ ვიცი

1. ექიმი ხშირად ნიშნავს ანტიბიოტიკს პაციენტის მოლოდინის მიხედვით

􀍙 ვეთანხმები

􀍙 არ ვეთანხმები

􀍙 არ ვიცი

1. ვენდობი ექიმის გადაწყვეტილებას ანტიბიოტიკების დანიშვნის საკითხზე.

􀍙 ვეთანხმები

􀍙 არ ვეთანხმები

􀍙 არ ვიცი

1. ექიმი აკმაო დროს უთმობს პაციენტისთვის ანტიბიოტიკების მიღება/გამოყენების წესების ახსნას.

􀍙 ვეთანხმები

􀍙 არ ვეთანხმები

􀍙 არ ვიცი

1. აფთიაქის პერსონალი ხშირად გიხსნით ანტბიოტიკების მირების წესებს.

􀍙 ვეთანხმები

􀍙 არ ვეთანხმები

􀍙 არ ვიცი

1. ექიმისგან დამოოუკიდებლად, მე ყოველთვის/ხშირად ვიცი თუ როდის მჭირდება ანტიბიოტიკების მიღება.

􀍙 ვეთანხმები

􀍙 არ ვეთანხმები

􀍙 არ ვიცი

1. ვენდობი ექიმის გადაწყვეტილებას ანტიბიოტიკების არ დანიშვნის საკითხზე.

􀍙 ვეთანხმები

􀍙 არ ვეთანხმები

􀍙 არ ვიცი

1. ექიმი არის არაკომპეტენტური თუ ის არ ნიშნავს ანტიბიოტიკს მაშინ როდესაც პაციენტი თვლის რომ ანტიბიოტიკის დანიშვნა არის აუცილებელი.

􀍙 ვეთანხმები

􀍙 არ ვეთანხმები

􀍙 არ ვიცი
